# Recent Advances in Piezoelectric Compliant Devices for Ultrahigh-Precision Engineering

**DOI:** 10.3390/mi15121456

**Published:** 2024-11-29

**Authors:** Zeyi Wu, Zehao Wu, I-Ming Chen, Qingsong Xu

**Affiliations:** 1 Department of Electromechanical Engineering, Faculty of Science and Technology, University of Macau, Avenida da Universidade, Taipa, Macau, China; yc37930@um.edu.mo (Z.W.); zehaowu@um.edu.mo (Z.W.); 2 School of Mechanical and Aerospace Engineering, Nanyang Technological University, Singapore 639798, Singapore; michen@ntu.edu.sg

**Keywords:** precision engineering, piezoelectric transducer, compliant mechanisms, piezoelectric compliant device

## Abstract

With advancements in small-scale research fields, precision manipulation has become crucial for interacting with small objects. As research progresses, the demand for higher precision in manipulation has led to the emergence of ultrahigh-precision engineering (UHPE), which exhibits significant potential for various applications. Traditional rigid-body manipulators suffer from issues like backlash and friction, limiting their effectiveness at smaller-scale applications. Smart materials, particularly piezoelectric materials, offer promising solutions with their rapid response and high resolution, making them ideal for creating efficient piezoelectric transducers. Meanwhile, compliant mechanisms, which use elastic deformation to transmit force and motion, eliminate inaccuracies induced by rigid-body mechanisms. Integrating piezoelectric transducers and compliant mechanisms into piezoelectric compliant devices enhances UHPE system performance. This paper reviews the recent advances in piezoelectric compliant devices. By focusing on the utilization of piezoelectric transducers and compliant mechanisms, their applications in perception, energy harvesting, and actuation have been surveyed, and future research suggestions are discussed.

## 1. Introduction

With the development in the field of small-scale research, such as microscopy, micro-assembly, and cell-surgery, precision manipulation has become an essential ability for interacting with small objects [1,2,3]. As the relevant research deepens, the precision of manipulation faces higher requirements [4,5]. Therefore, there is a growing trend towards ultrahigh-precision engineering (UHPE), which has attracted significant attentions due to its broad application prospects. However, the conventional manipulators, which rely on rigid-body mechanisms such as rods and gears, exhibit several inherent drawbacks to achieving high precision [6,7], e.g., backlash (which is caused by gaps in part connections) and friction (which leads to wear and tear). These drawbacks significantly degrade motion accuracy and energy efficiency. Such effects are more prominent at smaller scales and thus limit the further development of rigid-body mechanism tools in UHPE. Therefore, it is desirable to develop new operation tools based on alternative principles that are appropriate for the scenarios with ultrahigh-precision requirements.

Smart material (SM) responds to external stimuli (such as, heat, electricity, and force), which has the ability to directly convert energy from one form to a mechanical one, and vice versa [8,9,10]. It is ideal for addressing the drawbacks of rigid-body mechanisms. Over the past century, a variety of smart materials have been discovered, including piezoelectric materials (PMs), thermoelectric materials, and magnetostrictive materials. These materials enable the development of more efficient, reliable, and compact devices. Among these SMs, PMs have been widely adopted due to their rapid response, large force output, high stiffness, and high precision. They can generate an internal electrical charge from an applied mechanical stress, and vice versa, known as the direct and reverse piezoelectric effects, respectively [11,12,13]. These features enable PMs to be used to produce a high-performance piezoelectric transducer (PET) for various applications, such as piezoelectric actuator (PEA) [14,15], piezoelectric harvester (PEH) [16,17], and piezoelectric sensor (PES) [18,19].

At the same time, the compliant mechanism (CM), which continuously transmits force and motion through elastic deformation of the material, can overcome the inaccuracy induced by backlash and friction in rigid-body mechanisms [20,21,22,23]. The CM can also be designed as a monolithic structure to further reduce the assembly error. Moreover, its maintenance-free feature provides a higher reliability in practical use. These advantages have led to wide applications of CM in micromanipulation. In conjunction with PET, CM is commonly used for input or output amplification to address the performance limitations of PET [24,25,26]. As an integrated design of PET and CM, the piezoelectric compliant device (PECD) provides an effective tool in UHPE systems.

**Figure 1:**
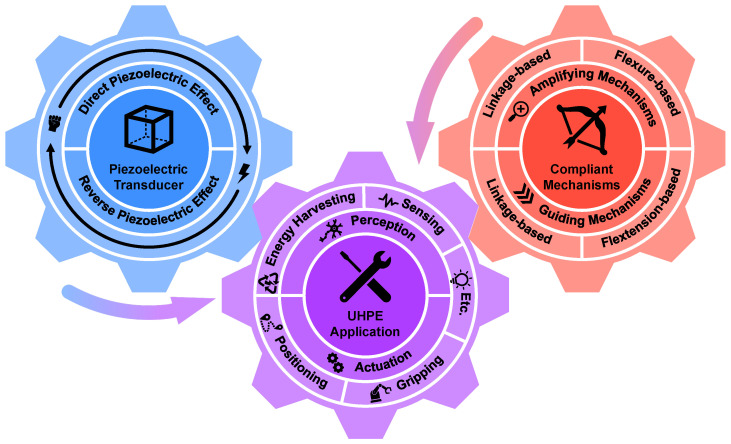
Overview of this review paper. The UHPE application is geared to PETs and CMs together.

This paper conducts a review of recent advances in PECDs for UHPE (see Figure 1), which exhibits the current state-of-the-art designs utilizing the special characteristics of PET and CM. The remaining parts of the paper are organized as follows. Section 2 presents the mechanisms and properties of PETs. Section 3 introduces the CM and its classification based on functions, including the principles and common designs. Section 4 outlines their applications in various fields, involving perception, energy harvesting, and actuation. Section 5 discusses the tendency of the related research and concludes the paper.

## 2. Piezoelectric Transducer

**Figure 2:**
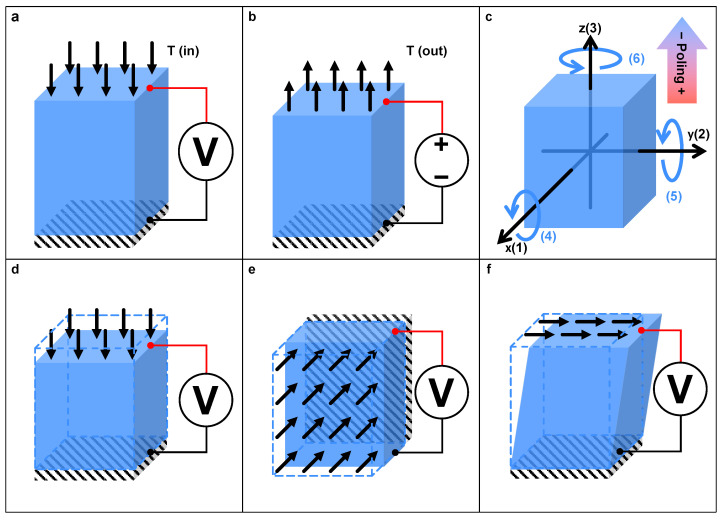
Schematic diagrams of piezoelectric materials. (**a**) Direct and (**b**) reverse piezoelectric effects. (**c**) The coordinate system of PMs based on the poling direction. Three working modes of PETs: (**d**) compression mode, (**e**) transverse mode, and (**f**) shear mode.

Through the past decades of research, several types of PMs have been discovered and developed extensively, which can be classified as crystalline, ceramic, and polymeric ones. They include crystalline materials (e.g., quartz and topaz), ceramics (e.g., barium titanate, lead titanate, lead zirconate titanate (PZT), macro fiber composites (MFC)), and polymers (e.g., polyvinylidene difluoride (PVDF) and polyvinylidene chloride (PVDC)). The main characteristic of these PMs is piezoelectricity. It is a phenomenon that materials generate an electrical potential when a mechanical stress is exerted. Referring to the schematic diagram in Figure 2a,b, the reversible electromechanical energy conversion processes, named direct and reverse piezoelectric effects, respectively, are the main working principle of PETs [27,28,29].

On the microscopic scale, the piezoelectric effect is caused by the change in polarization of anisotropic materials [30]. Some materials can be further enhanced by a poling process [31], i.e., applying an external electric field across the materials to align the electric dipoles. As shown in Figure 2c, the poling direction is commonly defined as the z-axis to build a right-handed coordinate system to facilitate describing the anisotropic properties of the material, where the direction and shear about the x-, y-, and z-axes are denoted by the subscripts from 1 to 6.

In practical applications, there are two important constants, i.e., the piezoelectric charge/strain constant *d* and voltage constant *g* (where *i* is the electric field direction, and *j* is the stress or strain direction), which are crucial indicators for evaluating the suitability of certain materials for either actuation or sensing application. These two constants have a relationship defined as $d=g\varepsilon^{T}$, where $\varepsilon^{T}$ represents the absolute permittivity under a constant stress field.

The piezoelectric constitutive equations [32,33,34], which mathematically describe the electromechanical relationship of PMs, are represented in the following.

(1) $$S_{ij}=\sum_{k,l}s_{ijkl}^{E}T_{kl}+\sum_{k}d_{kij}E_{k},$$

(2) $$D_{i}=\sum_{j,k}d_{ijk}T_{jk}+\sum_{j}\varepsilon_{ij}^{T}E_{j},$$

where *S*, *T*, *E*, *D*, and $s^{E}$ are strain, stress, electric field strength, electric flux density, and elastic compliance under a constant electric field, respectively (with i,j,k,l=1,2,3). Furthermore, by relabeling the subscripts in Voigt form (11→1; 22→2; 33→3; 23→4; 13→5; 12→6), Equations (1) and (2) can be expressed in the matrix form as follows.

(3) $$\left[\begin{array}{c} S_{1}\\ S_{2}\\ S_{3}\\ S_{4}\\ S_{5}\\ S_{6} \end{array}\right]=\left[\begin{array}{cccccc} s_{11}^{E} & s_{12}^{E} & s_{13}^{E} & 0 & 0 & 0\\ s_{21}^{E} & s_{22}^{E} & s_{23}^{E} & 0 & 0 & 0\\ s_{31}^{E} & s_{32}^{E} & s_{33}^{E} & 0 & 0 & 0\\ 0 & 0 & 0 & s_{44}^{E} & 0 & 0\\ 0 & 0 & 0 & 0 & s_{55}^{E} & 0\\ 0 & 0 & 0 & 0 & 0 & s_{66}^{E} \end{array}\right]\left[\begin{array}{c} T_{1}\\ T_{2}\\ T_{3}\\ T_{4}\\ T_{5}\\ T_{6} \end{array}\right]+\left[\begin{array}{ccc} 0 & 0 & d_{31}\\ 0 & 0 & d_{32}\\ 0 & 0 & d_{33}\\ 0 & d_{24} & 0\\ d_{15} & 0 & 0\\ 0 & 0 & 0 \end{array}\right]\left[\begin{array}{c} E_{1}\\ E_{2}\\ E_{3} \end{array}\right]$$

(4) $$\left[\begin{array}{c} D_{1}\\ D_{2}\\ D_{3} \end{array}\right]=\left[\begin{array}{cccccc} 0 & 0 & 0 & 0 & d_{15} & 0\\ 0 & 0 & 0 & d_{24} & 0 & 0\\ d_{31} & d_{32} & d_{33} & 0 & 0 & 0 \end{array}\right]\left[\begin{array}{c} T_{1}\\ T_{2}\\ T_{3}\\ T_{4}\\ T_{5}\\ T_{6} \end{array}\right]+\left[\begin{array}{ccc} \varepsilon_{11} & 0 & 0\\ 0 & \varepsilon_{22} & 0\\ 0 & 0 & \varepsilon_{33} \end{array}\right]\left[\begin{array}{c} E_{1}\\ E_{2}\\ E_{3} \end{array}\right]$$

Figure 2d,e illustrate three working modes of PETs, i.e., compression mode, transverse mode, and shear mode. These three modes are generally defined as 33-mode, 31-mode, and 15-mode in short, where the two numbers represent the directions of generated polarization and applied stress in turn. Depending on specific applications, the mentioned working modes can be paired with various types of structures of PETs to meet the requirements. For instance, unimorph- and bimorph-type PETs have a low stiffness and can endure a large deflection, but their output force is relatively small. These characteristics make them appropriate for force and strain sensing or actuation with free loads such as a micro-fan. Alternatively, stack-type PETs exhibit a high stiffness and a large output force, making them more appropriate for actuation and large force sensing applications [35]. Additionally, according to specific sites and applications, the PET can also be prepared in other specialized forms, such as fabric [36] and nanofiber [37], which are used in the field of piezoelectric flexible devices [38,39].

In the following sections, the important properties of PET are specified according to different utilization scenarios of the direct and reverse piezoelectric effects.

### 2.1. Direct Piezoelectric Effect

According to the direct piezoelectric effect, the PES can easily convert dynamic stress into an electric charge. Therefore, the PES can be adopted to convert physical signals into electrical signals, which are suitable for sensing and energy harvesting. For particular applications, the direction of applied stress mainly influences the conversion performance. The two common working modes of PES are 33-mode and 31-mode. Typically, the 33-mode excels in high voltage output, while the 31-mode is superior in high current output. Consequently, the open circuit voltage $V_{oc}$ of the PES is expressed as follows [40,41].

(5) $$V_{oc}=\frac{d_{ijk}}{\varepsilon_{ij}^{T}}T_{jk}g_{e}$$

where $g_{e}$ denotes the distance between the electrodes. For achieving a better output performance, the PES is suggested to be set at the location with larger strain, i.e., the terminal of a leaf flexure.

Furthermore, when considering a design utilizing a direct piezoelectric effect, the stiffness and fracture strain threshold of the selected PES mainly influence the sensitivity, working range, and maximum payload of the designed device. Therefore, the stiffness and fracture strain threshold of the PES should be considered when selecting suitable PES for sensing and energy harvesting scenarios.

### 2.2. Reverse Piezoelectric Effect

**Figure 3:**
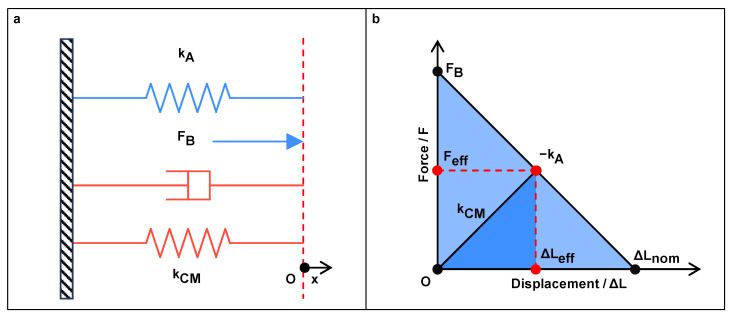
(**a**) Schematic diagram of a piezo-driven CM and (**b**) the relationship between force and displacement output of the PEA under maximum operating voltage.

The reverse piezoelectric effect is usually adopted for actuating purpose, which generates a mechanical output, such as force and displacement [42]. Regarding a specific PEA, its elastic compliance $s^{E}$ and piezoelectric charge/strain constant *d* are constant specifications determined by the material, structure, and poling treatment. Moreover, when an operating voltage is applied, the corresponding electric field strength *E* can also be determined. In this way, Equation (1) becomes a negatively correlated linear equation of strain *S* and stress *T*. From the perspective of the actuator, the linear relationship is reflected between its force and displacement output. Figure 3b illustrates this relationship under the maximum operating voltage of PEA. It reveals two important properties of PEA: nominal displacement $\Delta L_{\mathrm{nom}}$ without external force and blocking force $F_{B}$ under fully blocked condition. Their ratio $F_{B}/\Delta L_{\mathrm{nom}}$ is the output stiffness $k_{A}$ of the actuator. As a consequence, the equation of the curve can be expressed as follows.

(6) $$F=F_{B}-k_{A}\Delta L\;\Delta L\in \left[0,\Delta L_{\mathrm{nom}}\right]$$

Referring to Figure 3a, when considering the actuation of a CM that has an input stiffness of $k_{\mathrm{CM}}$, the output force of the actuator and reaction force generated by the CM will be balanced at a certain value at a particular displacement. They are defined as the effective force output $F_{\mathrm{eff}}$ and displacement output $\Delta L_{\mathrm{eff}}$ (see Figure 3b). Through the aforementioned force equilibrium condition, their expressions can be derived as follows.

(7) $$F_{\mathrm{eff}}=\frac{k_{\mathrm{CM}}}{k_{A}+k_{\mathrm{CM}}}F_{B}$$

(8) $$\Delta L_{\mathrm{eff}}=\frac{k_{A}}{k_{A}+k_{\mathrm{CM}}}\Delta L_{\mathrm{nom}}$$

These two equations can assist in the design process of a piezoelectric-driven CM. For example, they can be employed to set up the design objectives and constraints of CMs once given a PEA or select a suitable PEA to drive specific CMs to achieve the desired output displacement.

## 3. Compliant Mechanisms

CMs make use of the flexibility of materials (functioning through the deformation within a elastic region of materials) to transmit force and motion continuously. During the deformation process, the energy is stored in and subsequently released from the structure. In contrast with rigid-body mechanisms, CMs offer several advantages in terms of no backlash (ensuring the precise control and movement), frictionless process (avoiding the energy loss during the operation), and being maintenance free (eliminating the requirement of lubrication). Furthermore, CMs can be designed and manufactured as monolithic structures, which eliminates assembly errors and simplifies manufacturing processes. Hence, they can enhance the reliability and cost-effectiveness and allow more complex and compact designs. Therefore, CMs have been widely adopted in various fields, including precision manipulation and sensing in microelectromechanical systems (MEMS), biomedical applications, etc.

**Figure 4:**
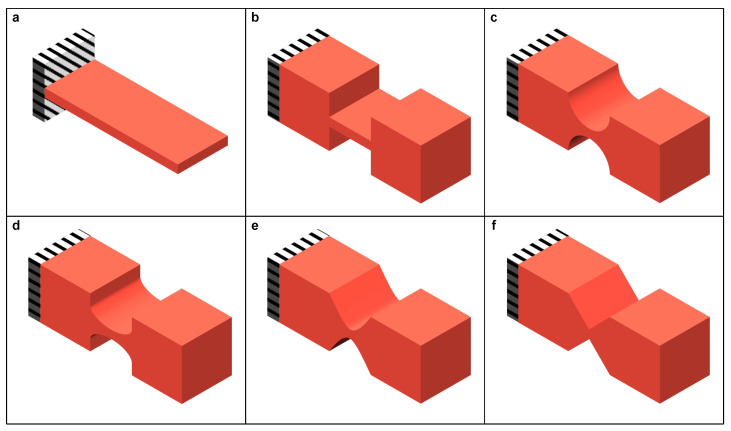
Typical compliant components. (**a**) Leaf flexure. Compliant hinges with notch shape of (**b**) rectangular, (**c**) semi-circular, (**d**) semi-elliptical, (**e**) parabolic, and (**f**) triangular type.

Concerning the structure of CMs, compliant components are the essential functional components that provide the ability of on-demand deformation for the structure. Therefore, their characteristics affect the final performance of CMs. Figure 4 illustrates the typical compliant components. Depending on the geometry and dimensions, different types of compliant components exhibit various characteristics. For example, the leaf flexures exhibit low stiffness in their working direction and can withstand a large deflection due to their distributed compliance. The compliant hinges with different shapes of notches, i.e., rectangular, semi-circular, and semi-elliptical types, demonstrating varying degrees of axial shift, stress concentration, and motion accuracy [43,44].

To describe and predict the behavior of a CM and optimize its dimensions to obtain the required performance, modeling and analysis are essential. Various modeling methods have been proposed in the literature, such as the pseudo-rigid-body method (PRBM) [45,46,47], compliance matrix method (CMM) [48,49,50], elliptic integral method (EIM) [51,52,53], chained beam constraint model (CBCM) [54,55,56], and finite element analysis (FEA). These methods have been extensively examined for their applicability and are well concluded and compared in the literature [57,58].

**Figure 5:**
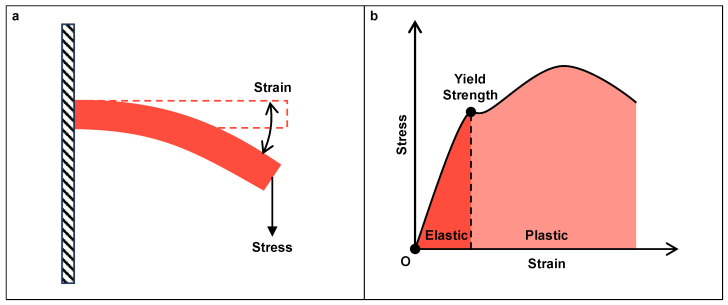
(**a**) Stress and strain of a bending flexure; (**b**) Typical stress–strain curve of the material and its elastic and plastic regions.

Since the elastic deformation process governs the working principle of CM, the material properties related to this process directly affect the performance of CMs. Generally, two material properties are considered for the selection, i.e., low Young’s modulus (which reduces the stress generated during deformation) and high yield strength (which allows the material to withstand larger stress before the deformation process becomes a plastic one) to experience a larger deformation or payload (see Figure 5b). Therefore, metallic materials, such as aluminum alloys, stainless steel, and titanium alloys, become common choices. Especially the aluminum alloy, which is widely used due to its high stiffness, low density, and cost efficiency.

Because of their similar characteristics of high precision and high energy efficiency, CMs are commonly used to enhance specific performance of PMs, e.g., amplifying force or displacement output and guiding the output direction to reduce lateral disturbance. In these two categories, various implementation methods have been proposed in the recent research, which are discussed in this section.

### 3.1. Amplifying Mechanisms

In some practical scenarios, PETs suffer from the problem of performance limitations. For instance, acting as an actuator, the stroke of stack-type PEA is only 0.1% to 0.2% of its length, making it hard to employ such an actuator to fulfill the stroke requirement [24,25,26]. In the scenario of sensing, the high stiffness of a stack-type PES results in small deformation and, hence, weakens the signal strength. To address the above problems rather than replacing them with higher-performance PETs, mechanical amplification is a more cost-effective approach to achieve the objective [35,59], such as amplifying the displacement output of PEA and the force input of PES. In this paper, the implementing methods of such amplifying mechanisms are classified as linkage-based and flextension-based ones.

#### 3.1.1. Linkage-Based Amplifier

The linkage-based amplifier (LBA) is a type of mechanical amplifier that utilizes linkage mechanisms with a specific mechanical advantage. Corresponding to its rigid-body counterpart, the compliant linkage adopts compliant hinges and linear actuators (or guiders) to replace the revolute and prismatic joints, respectively, which is known as the rigid-body replacement method. According to the quantity of links, the LBA can exhibit different configurations, which involve lever-type, bridge-type, pantograph-type, Scott–Russell-type, etc.

**Figure 6:**
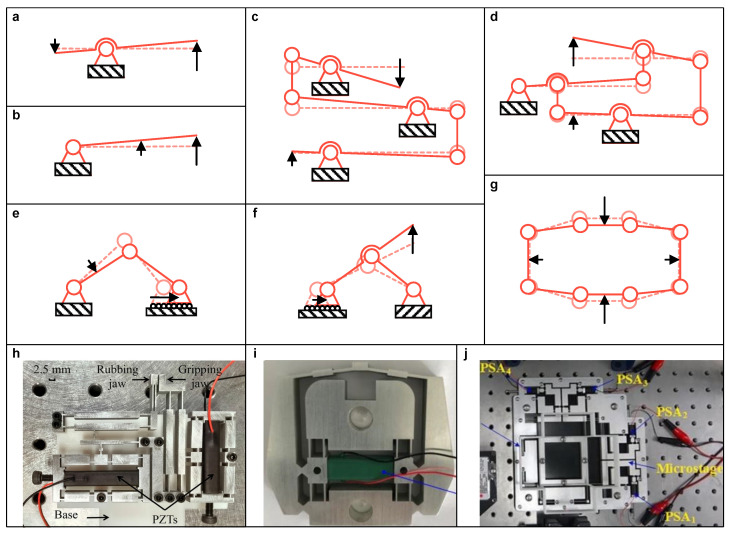
Typical types of linkage-based amplifiers. Lever-type with configuration of (**a**) class 1 and (**b**) class 3, (**c**) multi-stage, and (**d**) differential; (**e**) triangular-type, (**f**) Scott–Russell-type, and (**g**) bridge-type, where the triangle, circle, line, and arrow represent the fixed support, compliant joint, rigid link, and motion, respectively. The same applies below unless specified otherwise. Examples of design: (**h**) Bridge-type and lever-type. Reproduced with permission from [61], ©2023 IEEE. (**i**) Lever-type and half bridge-type. Reproduced under terms of the CC-BY license from [62]. (**j**) Four-bar linkage and lever-type. Reproduced with permission from [63], ©2021 Elsevier.

The compliant lever is composed of a compliant hinge and rigid link, which function as the pivot and arm, respectively. It exhibits an amplifying characteristic similar to that of a rigid-body lever. Figure 6a–d show a series of lever-type amplifiers. The amplification ratio is related to the length of the load and effort arm. Similarly, the lever-type amplifier can also be used to redirect the input direction by adopting a specific arm design and lever type. Due to its simple amplifying principle, the lever-type amplifier is widely used to achieve a high ratio of amplification. However, it results in a bulky size and efficiency loss due to the adoption of numerous compliant hinges. To increase the compactness and efficiency, other linkages with three or more links can be employed to inspire the design of LBA. For example, the triangular-type (see Figure 6e) and Scott–Russell-type (see Figure 6f) both refer to crank-slider four-bar linkage.

In the literature, the complementary design usually combines various LBAs to make a compromise among the required performances. For instance, Chen et al. [60] adopted two compound bridge-type amplifiers placed in the same output direction, which work together to drive a parallelogram mechanism with symmetrical forces to address the issue of parasitic motion for a parallelogram mechanism. Lyu et al. [61] utilized a two-stage lever-type amplifier to improve the rubbing stroke and integrated the last stage lever into a parallelogram mechanism to effectively reduce the parasitic displacement (see Figure 6h). Ling et al. [62] presented a two-stage amplifier that used the output of two symmetrical lever-type amplifiers as the input of a half-bridge-type amplifier to enhance the dynamic bandwidth of PEA (see Figure 6i). Yang et al. [63] introduced a double-rocker mechanism, which is a four-bar linkage with lever-type amplifiers connected at the input and output ends (see Figure 6j).

#### 3.1.2. Flextension-Based Amplifier

**Figure 7:**
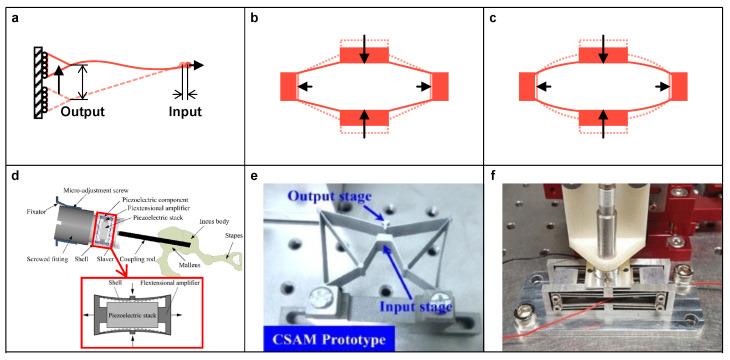
(**a**) Schematic diagram of flextension; Typical types of flextension-based amplifier: (**b**) rhombus-type and (**c**) ellipse-type; Examples of design: (**d**) Piezo-driven middle ear implant. Reproduced under terms of the CC-BY license from [65]. (**e**) Stroke amplifier with fully compliant structure. Reproduced under terms of the CC-BY license from [66]. (**f**) Sandwich-like vertical positioning stage. Reproduced with permission from [67], ©2019 IEEE.

Flextension-based amplifier (FBA) is another type of mechanical amplifier that utilizes the flexibility of a specific structure [64]. As shown in Figure 7a, when a horizontal input is applied at the free end of an inclined flexure, there is a vertical output at the other end whose magnitude depends on the applied input and the shape of the flexure. Thus, this principle can be utilized for the amplification of force and displacement. In comparison to LBA, the flexibility of FBA is reflected by its compliant links without hinges [24,64]. For instance, the rhombus-type amplifier is a typical FBA that has a similar structure to that of the bridge-type or triangular-type amplifier, but it does not have joints.

Due to the usage of compliant links, FBA is characterized by its distributed compliance, which alleviates the problem of stress concentration during operation. According to the shape of compliant links, FBAs can be classified as rhombus-type (see Figure 7b), ellipse-type (see Figure 7c), etc. For example, Liu et al. [65] adopted a reverse ellipse-type amplifier in a piezo-driven middle ear implant for amplifying the displacement of the PEA. It significantly decreases the power consumption of PEA to achieve the same output, and, therefore, extends the battery life (see Figure 7d). Li et al. [66] proposed a stroke amplifier with a fully compliant structure. It provided a high amplification ratio of more than 13, which adopted a half rhombus-type amplifier as the last stage (see Figure 7e). Moreover, Wu and Xu [67] introduced a spatial kind of reverse rhombus-type amplifier for vertical positioning, which is a sandwich-like structure for separating the amplifiers and PEA in three planes. It reduces the stage height and leads to a compact dimension (see Figure 7f).

### 3.2. Guiding Mechanisms

The compliant hinge, which acts like a revolute joint with a limited range, is the main component of the CMs. However, the rotation axis of a compliant hinge exhibits varying degrees of shifting during rotation, depending on the shape of hinge [68,69]. This variation causes the deviation in motion direction. Furthermore, manufacturing and assembly tolerances can also induce further deviation in practice. Additionally, interaction with an external object or environment may introduce a lateral reaction to the CM, misguiding the motion. Therefore, to maintain the motion direction and isolate undesired disturbance, it is necessary to integrate a guider into the CM design. In the case of a multi-degree-of-freedom (multi-DOF) device, compliant guiders can be utilized as decouplers to isolate the dominant motion from other axes. In this paper, the compliant guiders are classified into linkage-based and flexure-based ones.

#### 3.2.1. Linkage-Based Guider

**Figure 8:**
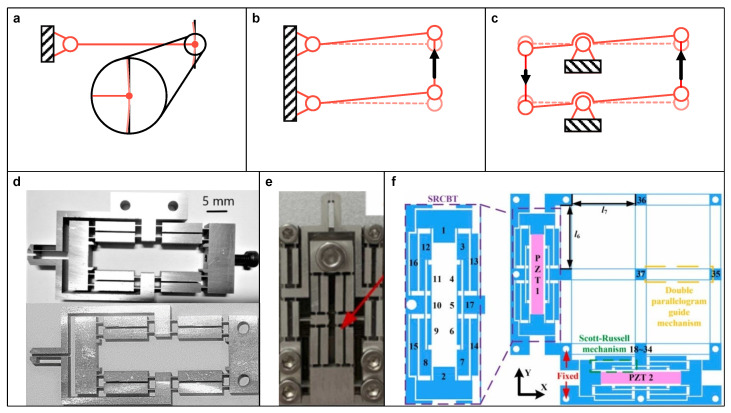
Schematic diagram of linkage-based guider. (**a**) Approximate linear motion of a hinged-free beam, (**b**) the parallelogram-type, and (**c**) the compound lever-type; Examples of design: (**d**) Compound bridge-type. Reproduced with permissions from [70,71], ©2020 Springer Nature and ©2020 IOP Publishing, respectively. (**e**) The combination of compound bridge-type, compound lever-type, and parallelogram-type. Reproduced with permission from [75], ©2024 Elsevier. (**f**) Bridge-type embedded with Scott–Russell mechanism. Reproduced with permission from [76], ©2021 Elsevier.

Similar to the aforementioned LBA, the design of a linkage-based guider (LBG) can be realized by referring to perfect or approximate straight-line linkages like the Scott–Russell linkage. Regarding a CM (such as a rigid-body beam) connecting to a fixed compliant hinge, as illustrated in Figure 8a, because the allowable angular deflection of the compliant hinges is relatively small, the free terminal of the rigid-body link only produces a short arc trajectory and can be considered approximately linear. Thus, those circular motion linkages are also referable for CM. For example, Figure 8b shows a parallelogram-type LBG, which has better directionality due to its inherent yaw motion constraint.

As some LBAs offer directed output, they can also be regarded as a special type of LBG. In practice, it is common to further improve the directionality of these LBGs by utilizing a compound configuration, like the compound lever-type LBG, as shown in Figure 8c. These designs implement both amplifying and guiding functions via a compact structure, which are widely adopted in the literature. For instance, Das et al. [70,71] utilized a compound bridge-type LBG to achieve gripping with low parasitic motion (see Figure 8d). Zhao et al. [72] and Lyu et al. [61,73,74] both adopted a compound lever-type LBG with a single input to guide gripping jaws and reduce the parasitic angular motion. Chen et al. [75] proposed a piezoelectric microgripper with both parallelogram-type and compound bridge-type LBG, which further reduce the parasitic linear motion (see Figure 8e). Wu et al. [76] employed the Scott–Russell mechanism to replace one arm of the compound bridge-type LBG, aiming to improve its lateral stiffness and natural frequency in the working direction (see Figure 8f).

#### 3.2.2. Flexure-Based Guider

**Figure 9:**
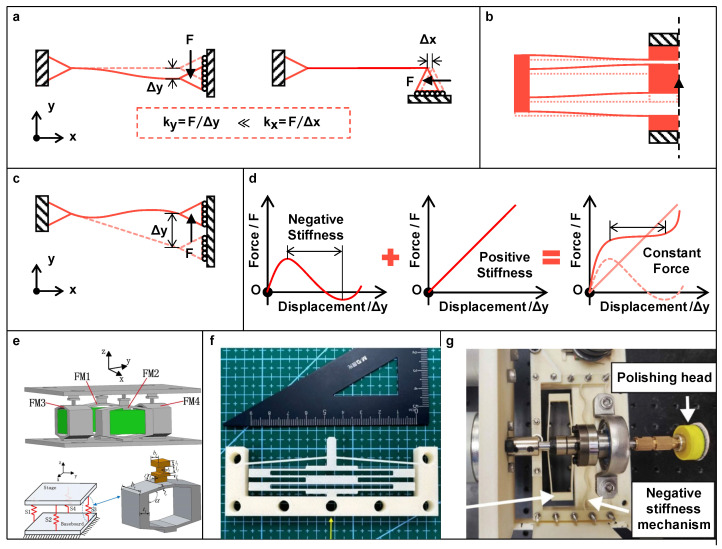
(**a**) Schematic diagram of a fixed-guided leaf flexure under vertical and horizontal load; (**b**) The two-stage compound parallelogram flexure guider; (**c**) Schematic diagram of an inclined flexure that provides negative stiffness; (**d**) The force-displacement relationship of the negative- and positive-stiffness mechanism and their superimposed; Examples of design: (**e**) The spatial 2-DOF decoupler for guiding and decoupling the tilt motion. Reproduced with permission from [78], ©2019 Elsevier. (**f**) CFM for salmon egg manipulation. Reproduced with permission from [81], ©2024 Elsevier. (**g**) CFM for polishing. Reproduced with permission from [82], ©2024 IEEE.

Concerning a single-DOF compliant structure, such as a fixed-guided leaf flexure, as illustrated in Figure 9a, it exhibits different stiffness values depending on the direction of load exerted at its guided terminal, which can be utilized for motion guiding. In practice, a pair of mirror-symmetric leaf flexures can be considered as the simplest flexure-based guider (FBG). As the basic unit of its category, leaf flexures can be superimposed by multiple units in various configurations to achieve a specific performance. For example, Figure 9b shows a two-stage compound parallelogram flexure guider, which enhances the restriction of yaw and pitch motion while maintaining the input stiffness.

Compared to LBG, the FBG is characterized by a distributed compliance, which allows a relatively larger deflection. Thus, FBGs exhibit a greater agility in various scenarios. In the literature, Xun et al. [77] designed a spatial screw compliant mechanism based on multiple helically arranged flexures, which can transmit a linear motion into rotary motion. Shao et al. [78] connected two leaf flexures in series (orthogonally in two planes) to obtain a spatial 2-DOF decoupler and employed it for guiding and decoupling the tilt motion in two directions of a piezo-driven orientation stage (see Figure 9e).

On the basis of LBG, constant force mechanisms (CFMs) have been introduced that exhibit a constant reaction force and quasi-zero stiffness within a specific motion region [79]. To have a high guiding performance (i.e., with near-zero input stiffness while maintaining a high lateral stiffness), CFMs can be designed by a direct zero-stiffness configuration. Alternatively, it can be devised by superimposing a positive-stiffness and a negative-stiffness mechanism, where the negative stiffness is obtained by the buckling state of the structure or special flexure designs. Figure 9c illustrates the schematic diagram of an inclined flexure that provides negative stiffness in a specific motion range. The solid curves in Figure 9d depict the force-displacement relationships of the negative- and positive-stiffness mechanisms and their superimposed result. Within the constant-force region, in addition to the usage in guiding, the adoption of CFMs can reduce the actuation force for the same strain and limit the force applied to the target object. Based on this characteristic, researchers have proposed various designs for specific tasks. For example, Ding et al. [80] presented a quasi-zero stiffness vibration isolator composed of three sets of CFMs for low frequency vibration isolation. Lyu and Xu [81] designed a CFM and validated its feasibility in manipulating salmon eggs (see Figure 9f). Wu et al. [82] and Wei and Xu [83] introduced CFMs to the tool head design for implementing constant-force robotic polishing (see Figure 9g).

## 4. Integrated Applications

Taking advantage of the reversible piezoelectric effect of PETs and the continuous force and motion transmission of CMs, their integration into PECDs has been adopted in the UHPE system for various applications, such as high-resolution sensing, high-efficiency energy harvesting, and high-precision actuation. In this section, recent advances in these applications across various fields are introduced, highlighting the differences that vary according to design objectives.

### 4.1. Perception and Sensing

**Figure 10:**
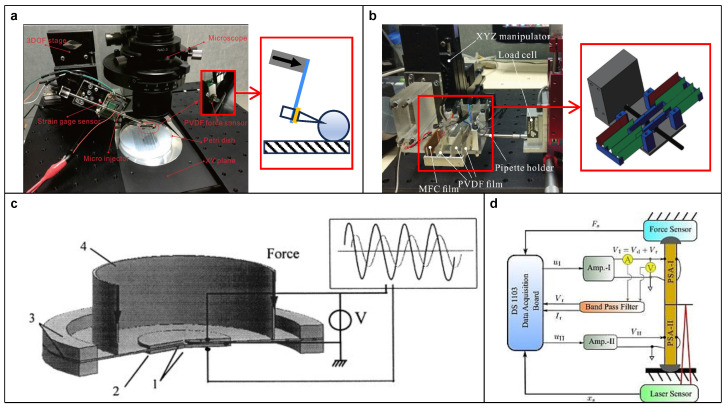
(**a**) A PVDF film integrated onto an injection micropipette for cell piercing. Reproduced with permission from [93], ©2017 IEEE. (**b**) A two-stage compound parallelogram flexure guider constructed from PVDF and MFC films for force sensing. Reproduced with permission from [94], ©2017 IEEE. Static force PES based on: (**c**) Structure resonant method. Reproduced with permission from [95], ©2000 Elsevier. (**d**) Capacitance measurement. Reproduced with permission from [96], ©2018 Elsevier.

Based on the direct piezoelectric effect, the PES can detect its own dynamic deformation or that of an attachment, which can be utilized to achieve tactile perception. In addition to visual perception, the tactile perception has gained significant attention [84,85,86,87,88], especially in dexterous tasks where accurate and timely tactile feedback is crucial for achieving flexible interaction with target objects [89,90,91,92]. For instance, to reduce cell injury during the cell piercing process, Wang and Xu [93] proposed a piezo-driven microinjection system by integrating a PVDF film into the injection micropipette for force sensing (see Figure 10a). They used a store-and-hold charge amplifier device to convert the generated charge into a static voltage signal. Thereby, the force during cell piercing process was clearly measured. However, the cantilever structure of the PVDF film setup is easily affected by external disturbances, resulting in inaccurate force sensing. To address this defect, Wei and Xu [94] proposed a new design using a two-stage compound parallelogram flexure guider, where the flexures were constructed by PVDF and MFC films (see Figure 10b). The setup ensures that the films can deform as expected and correctly reflect the force applied in a certain direction.

Due to the charge leakage, the above direct measurement, or so-called non-resonant method, is only applicable to dynamic force sensing, such as the piercing process. In case of static or ultra-low frequency force measurement, the resonant method is more appropriate. It works based on the phenomenon that altering the boundary conditions of a structure, such as exerted force, will lead to changes in its resonance characteristics, which can be mapped and utilized for sensing. For instance, Gehin et al. [95] introduced a PES composed of two piezoelectric disks attached to a steel diaphragm (see Figure 10c). One of the piezoelectric disks was used to excite the diaphragm to its first resonant frequency, while the other detected resulting vibrations. When a external force is exerted on the diaphragm, the resonant frequency of the structure is modified, and this change can be mapped to the applied force amplitude.

In addition to analyzing the electrical signal converted by the PES, static force measurement can also utilize the physical property changes of the PES itself. There are several methods based on electrical admittance [97], decay time constant [98], and capacitance [96,99] (see Figure 10d). Specifically, the electrical admittance and decay time constant methods are similar to the resonant method, because they also require the analysis of time-series data and share the same application scenarios. Although the capacitance method can be operated in real time, due to the small capacitance of the film-type PES and high stiffness of the stack-type PES, it is only applicable to the latter case where the applied force has a large magnitude or is amplified [100,101,102].

### 4.2. Energy Harvesting

Due to the principle of the direct piezoelectric effect, PM can be regarded as an energy converter that can convert mechanical energy into electrical energy for usage. With peripheral equipment such as rectification circuits, the PET can be used as an energy harvester that is able to power wireless sensors or directly serve as a passive sensor [103]. From another perspective, the research on improving the harvesting efficiency also benefits the enhancement of the sensitivity to specific type of signals, which is critical for the environmental perception and compensation of UHPE.

**Figure 11:**
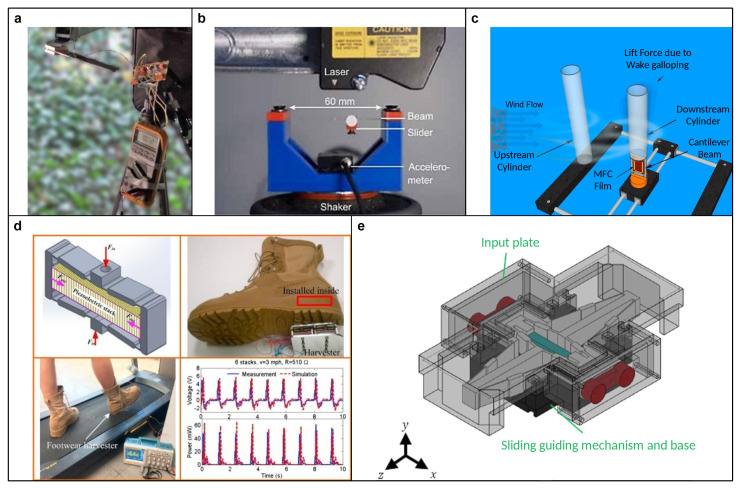
(**a**) A dedicated PEH for powering wireless health monitoring sensor nodes of traffic-induced bridge. Reproduced with permission from [107], ©2013 IOP Publishing. (**b**) PEH with self-tuning harvesting frequency through a movable slider. Reproduced with permission from [109], ©2013 Elsevier. (**c**) PEH based on the wake galloping phenomenon. Reproduced with permission from [122], ©2018 Elsevier. (**d**) A footwear energy harvester using six rhombus-type force amplifiers connected in parallel. Reproduced with permission from [131], ©2018 Elsevier. (**e**) Multi-directional PEH using a single piezoelectric stack. Reproduced with permission from [133], ©2021 IEEE.

For ubiquitous vibration energy, the most common PEH is based on the forced vibration of the cantilever beam [104,105,106], which has a particular resonant frequency according to the structure. To achieve a higher energy exchange efficiency, the resonant frequency of a cantilever-type PEH should be close to the ambient vibration frequency. For instance, Peigney and Siegert [107] measured the vibration frequency of a traffic-induced bridge and then designed a dedicated cantilever-type PEH for powering wireless health monitoring sensor nodes with low cycle duty (see Figure 11a). Yang et al. [108] adjusted the structure of a cantilever-type PEH to match its first and second resonant frequencies to those of the rail for powering trackside monitoring sensors. However, if the ambient vibration frequency varies, the efficiency of PEHs significantly decrease due to their narrow harvesting frequency bandwidth. To address this issue, researchers have proposed various solutions to broaden the harvesting frequency spectrum. For example, Miller et al. [109], Somkuwar et al. [110], and Jackson et al. [111] utilized a movable slider (see Figure 11b), roller, and liquid-filled box as the proof mass, respectively. These designs feature a self-tuning center of gravity that can be adjusted according to the excitation, thereby achieving a self-tuning harvesting frequency. Moreover, nonlinear monostable and multistable structures can effectively improve the harvesting frequency spectrum. For example, an impact-induced broadband vibration energy harvesting can be achieved by placing a stopper at a specific position under the beam [112,113]. The multistable coherent resonance can be achieved by utilizing multiple magnets to introduce the multistable characteristic to a cantilever-type PEH [114,115] or by employing a bistable structure based on the buckling effect [116,117].

To harness flow energy from wind, researchers have utilized its characteristics to design PEHs. For example, magnets carried on the blown fan blades can be used for periodic excitation [118,119,120]. Wu et al. [121] investigated vortex shedding frequency and designed a compact cantilever-type PEH capable of generating up to 1.02 W of electric power with wind speeds of 9–10 m/s. Usman et al. [122] proposed a system for a broad wind spectrum using the wake galloping phenomenon (see Figure 11c). To capture energy from slow wind speeds, Kwon [123], Wang [124], and Liu [125] explored T-shaped, Y-shaped, and fork-shaped piezoelectric cantilevers, respectively. These designs are also applicable to water flow energy harvesting [126,127].

The mechanical energy generated by human activities, such as heart motion and walking [128], has also garnered significant attention from researchers. Most PEHs that capture energy from heart motion are used to power pacemakers [129]. The research in this area is similar to vibration energy harvesting, focusing on matching the heartbeat frequency [104,130]. Due to their small deflection and high energy density, piezoelectric stacks are commonly used to harvest energy from human walking. They minimally impact the walker and can generate substantial energy per step. Common types of PEHs include footwear and underfloor harvesters. For example, Qian et al. [131] designed a footwear energy harvester using six rhombus-type force amplifiers connected in parallel (see Figure 11d). Shubham et al. [132] developed a shoe-pad harvester that maintains flexibility even with an array of piezoelectric stacks. These footwear harvesters are effective solutions for powering implantable biomedical devices. On the other hand, underfloor harvesters are typically installed in high-traffic areas (e.g., school entrances, hospitals, and customs), which impose fewer space constraints. They can utilize a single piezoelectric stack to capture multi-directional forces (i.e., pressure and friction by walking), making them more economical and efficient. Xu’s group [133,134,135,136] proposed several multi-directional energy harvesters using a single piezoelectric stack (see Figure 11e).

### 4.3. Actuation

The PECDs for actuation utilize the reverse piezoelectric effect of PEAs as the actuating source and CMs as the functional component to achieve active manipulation of target objects. In general, the demands of manipulation focus on two operations: moving and holding, which fulfill requirements in various fields, such as microassembly, biomedical applications, microscopy, etc.

#### 4.3.1. Positioning

The positioning operation involves moving the end-effectors or target objects to the desired location. The workspace and operating speed are two indicators used to evaluate the performance of a positioning stage. Additionally, for a multi-DOF positioning stage, the number of DOFs represents the flexibility in micromanipulation, where each additional DOF further enriches the application scenarios. Common multi-DOF positioning stages include XY, XYθ, and XYZ stages. Their implementation of multi-DOF output involves two configurations: serial and parallel kinematics.

**Figure 12:**
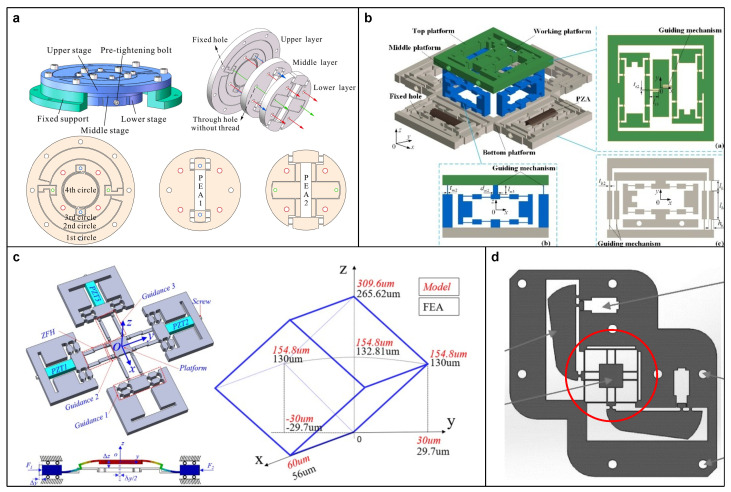
(**a**) A dual-range rotation positioning stage composed of two sets of double slider crank mechanisms. Reproduced with permission from [139], ©2021 Elsevier. (**b**) A 6-DOF positioning stage composed of three substages in series. Reproduced with permission from [140], ©2018 Elsevier. (**c**) An XYZ parallel positioning stage with a compact and planar profile. Reproduced with permission from [141], ©2020 Springer Nature. (**d**) A widely used decoupling scheme for an XY positioning stage. Reproduced with permission from [144], ©2023 Elsevier.

The serial-kinematic configuration is a simple method for implementing a multi-DOF output by connecting the fixing terminal of a subsequent substage to the output end of a preceding one. In such a configuration, each substage remains relatively independent, allowing for the combination of distinct functionalities within a single system. In the literature, the serial-kinematic configuration is commonly employed for developing dual-stage systems, which are composed of two substages with the same number of DOFs, typically one for long stroke and the other for high resolution. For instance, Xu [137] designed a dual-stage positioning system featuring a 10 mm stroke and 500 nm resolution. It is composed of two positioning stages in serial, which are driven by a voice coil motor and a PEA, respectively. Ekbatani et al. [138] presented a fully piezo-driven dual-stage by adopting a compound bridge-type amplifier in the first substage with an amplified stroke of 648 µm and a high stiffness direct-driven substage with a resolution of 1 µm. Zhang et al. [139] designed a dual-range rotation positioning stage that is composed of two sets of double slider crank mechanisms (see Figure 12a). Such mechanisms employ compliant hinges for achieving high-resolution displacement output and leaf flexures for large stroke output, respectively. Simulation studies have identified two distinct output ranges of the stage, i.e., 0.88 mrad and 17.40 mrad.

However, in a serial-kinematic configuration, each subsequent substage inherits a cumulative inertial mass from the preceding ones, resulting in a different dynamic performance for each substage. This drawback also restricts the maximum number of DOFs that can be integrated into the stage. For example, Lin et al. [140] obtained a 6-DOF positioning stage by composing three substages in series, where the first substage carries the linear motion in the X- and Y-axes; the second one realizes the rolling motion, pitching motion, and linear motion in the Z-axis; and the last one implements the yaw motion (see Figure 12b). To mitigate the adverse effects of cumulative inertial mass, the design principles emphasizing compactness and reduced weight have also been proposed. For instance, Wu and Xu [67] designed a compact vertical positioning stage with the objective of reducing the additional inertial mass when connected in series to an XY positioning stage, thereby ensuring the dynamic performance in the XY plane.

In contrast to the serial-kinematic configuration, the parallel-kinematic configuration connects all substages in parallel, and each substage can directly drive the common output end. Benefiting from this feature, the stage can achieve consistent dynamic performance by adopting a symmetrical design with the required DOF. Moreover, the parallel-kinematic configuration can be further divided into coupled and decoupled types, whose difference lies in whether the output of each substage is decoupled. Specifically, a coupled-type stage can be treated as a multi-input multi-output (MIMO) system. While it requires complex kinematic modeling and advance control algorithms to manage the cross-talk between substages, it exhibits several advantages, such as high stiffness, large load capacity, and rapid response. For instance, Xie et al. [141] presented an XYZ parallel positioning stage with a compact and planar profile, which achieves 3-DOF output by adopting Z-shape flexure hinges to connect the actuating part and the platform (see Figure 12c).

On the other hand, the decoupled-type stage utilizes the decoupling mechanisms or decouplers to isolate the output motion among substages, thereby dividing the system into multiple single-input single-output (SISO) systems. Because the decoupler reduces the cross-talk between substages, it is easier to identify and compensate for the errors or nonlinearities in each axis, such as the hysteresis of PEA [142]. Therefore, the performance of a decoupled-type stage is highly dependent on the effectiveness of the motion decoupler, to which most of the improvement is attributed. For instance, Figure 12d shows a widely used decoupling scheme for an XY positioning stage [143,144]. The scheme involves four identical planar 2-DOF decouplers arranged symmetrically, where each decoupler functions as both guiding and lateral decoupling. Zhang et al. [145] improved the lateral decoupling of each axis by employing compound parallelogram mechanisms. Wang et al. [146] and Wu et al. [147] replaced all the leaf flexures with double parallelogram mechanisms, which enhances the guiding ability and bearing capacity of the decoupler.

#### 4.3.2. Gripping

**Figure 13:**
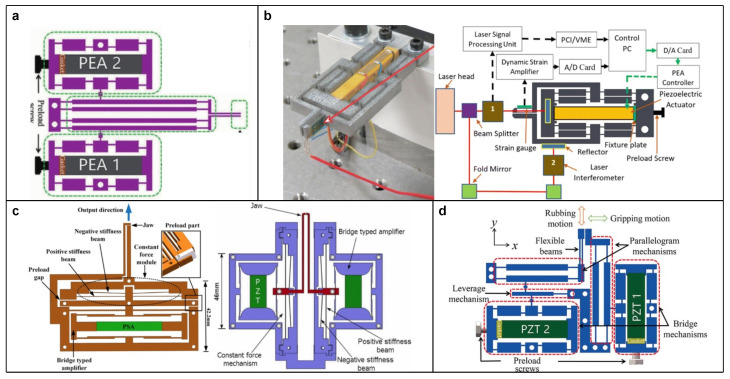
The microgrippers with: (**a**) Normally open and normally close jaws. Reproduced with permission from [73], ©2021 IEEE. (**b**) Active-type force control. Reproduced with permission from [70], ©2020 Springer Nature. (**c**) Passive-type force control. Reproduced with permission from [150] and [151], ©2017 IEEE and ©2016 IEEE, respectively. (**d**) Gripping and rubbing functions. Reproduced with permission from [61], ©2023 IEEE.

The gripping operation is to temporarily hold the target objects to facilitate subsequent operations. In principle, the gripping operation is the relative positioning of the gripper jaws. Hence, it has similar performance indices to the positioning operation, such as the stroke and speed of jaws, which determine the gripping range and efficiency, respectively [148]. For example, Chen et al. [149] developed a gripper with a high amplification ratio of 52 implemented by a two-stage amplification, and a maximum output displacement of 900 µm. Lyu and Xu [73] adopted two sets of actuating modules to drive two jaws in the same direction independently (see Figure 13a). Hence, the gripper not only has two working modes, i.e., normally open and normally close, but also achieves a large gripping range of 54.44–862.2 µm.

During the operation, grippers are required to provide sufficient gripping force to produce enough friction force between the jaws and target object. However, the gripping force should not be too excessive, as it may cause damage to delicate target objects, such as cells and fibers. Therefore, maintaining an appropriate gripping force is critical for these tasks. One solution is integrating force sensors into the system and achieving desired force output through feedback control. This active-type method, as mentioned in the previous section, had been widely adopted. For example, Zhao et al. [72] and Das et al. [70] both integrated strain gauges as force sensors on the flexible gripping jaws for precise force control (see Figure 13b).

The other solution is a passive type that combines CFM into the mechanical design of grippers. Within the designed constant force region, undesired factors such as overshoot control output and external disturbance are isolated by CFM, keeping the contact force near constant and thus protecting the target object. The related research mainly focuses on the motion region size of constant force. Liu et al. [150,151] introduced two different grippers that provide a constant force of 1.1 N in the 200 µm range and 530 mN in 220 µm range, respectively (see Figure 13c). On the other hand, as different tasks have various requirements for contact force, the adjustable feature of constant force could expand the scope of application. For instance, Gan et al. [152] designed an adjustable preloading mechanism for the CFM of a gripper, achieving a variable constant force ranging from 0.110 N to 1.735 N. Similarly, He et al. [153] adopted a bridge-type amplified PEA as the adjusting module to achieve online adjustment of the constant force magnitude. Zhang and Yan [154] added a bolt to one of the gripper jaws to make its gap adjustable, thus expanding the size interval of the objects that can be manipulated with a constant force.

Furthermore, concerning the scenarios where a single target object needs to be rotated, an ordinary gripper with a single-DOF needs to either rotate itself or the entire carrying platform around the target object after the gripping operation, which is inefficient and complicated to implement. Therefore, a 2-DOF gripper is a more realistic solution. By adding another translational DOF to the jaws, the gripper can implement a rubbing operation. For example, Lyu et al. [61] proposed a 2-DOF gripper whose two jaws are actuated independently that achieves gripping and rubbing strokes of 251.2 µm and 225.0 µm, respectively (see Figure 13d).

## 5. Conclusions

Through the literature review, a research trend can be identified that the majority of PECD designs emphasize reducing the physical compactness of the devices. The comparison of performance is generally based on a particular output over the occupied area or volume. This tendency can be attributed to the requirements of high compatibility, modularity, and cost effectiveness. Smaller devices offer significant advantages when integrated with existing equipment, especially in the MEMS, medical, and aerospace fields. For instance, when operating under a microscope with limited space, a compact micromanipulator is less likely to obstruct visibility or interfere with other equipment, thereby enhancing compatibility and avoiding the need to replace the main equipment. Additionally, compact designs increase the modularity of devices, making it easier to compose them for various applications, which enhances reusability. Moreover, small-sized devices consume less material and require less processing time, resulting in lower production costs and further reduced usage costs.Thus, the miniaturization of PECDs will become a major focus of future research, facilitating its wider application in UHPE systems. To achieve such an objective, the investigation on PET and CM (i.e., two main components of PECD) will be mainly propelled.

According to different functional requirements, the structure design and layout arrangement of PECD vary. However, its compactness is mostly constrained by the size of PET. One solution is to improve the piezoelectric performance of PET. For instance, Wang et al. [155] investigated the piezoelectricity modulation mechanism of Y-doped ZnO at the atomic scale, and the prepared sample demonstrates an 8.5-fold improvement in output performance. Ao et al. [156] used a temperature-pressure dual-field regulation method to construct an oriented tertiary structure in MXene/PVDF nanocomposite. This method enhances the piezoelectricity and improves the current output by nearly 23 times. Using advance PETs not only reduces the size while maintaining the required performance but also minimizes the need for additional structural design adjustments to match the size of PET, making the design of PECD more flexible and compact-oriented.

On the other hand, the static and dynamic characteristics, as well as the safety factor of compliant components, are closely related to their physical dimensions. Therefore, miniaturization will significantly influence the performance of PECDs, which requires compensation designs. Specifically, due to manufacturing limitations, achieving a metal component thinner than 0.5 mm is challenging. Such constraint means that the miniaturization of CM will result in relatively decreased compliance. To address such an issue, in addition to using PET with better performance, using CFM to reduce the stiffness can also be considered to enhance the final performance. In the literature, some designs can even reduce the reaction force to near zero [157,158]. Conversely, the affected dynamic performance can be enhanced by passive damping approaches [159], which involve attaching damping layers to the compliant components to improve linear dynamics.

In summary, this paper reviews the recent integration designs of PET and CM for UHPE applications. PMs exhibit direct and reverse piezoelectric effects, which enable the constructed devices various usages, such as PES, PEH, and PEA. Their important properties are also highlighted for selection reference. CMs are classified based on their functions and structures, which include amplifying and guiding mechanisms. The applications of PECD integrating PET and CM are summarized in three aspects: sensing, energy harvesting, and manipulation. Finally, the survey is concluded by predicting the trend of PECD miniaturization, highlighting the key research area for future development.

## Data Availability

No new data were created or analyzed in this study. Data sharing is not applicable to this article.

## Abbreviations

The following abbreviations are used in this manuscript:

UHPE	Ultrahigh-precision engineering
SM	Smart material
PM	Piezoelectric material
PET	Piezoelectric transducer
PEA	Piezoelectric actuator
PEH	Piezoelectric harvester
PES	Piezoelectric sensor
CM	Compliant mechanism
PECD	Piezoelectric compliant device
PZT	Lead zirconate titanate
MFC	Macro fiber composites
PVDF	Polyvinylidene difluoride
PVDC	Polyvinylidene chloride
MEMS	tMicroelectromechanical systems
PRBM	Pseudo-rigid-body method
CMM	Compliance matrix method
EIM	Elliptic integral method
CBCM	Chained beam constraint model
FEA	Finite element analysis
LBA	Linkage-based amplifier
FBA	Flextension-based amplifier
DOF	Degree-of-freedom
LBG	Linkage-based guider
FBG	Flexure-based guider
CFM	Constant force mechanism
MIMO	Multi-input multi-output
SISO	Single-input single-output
